# The Hospital Nursing Supplement

**Published:** 1893-06-17

**Authors:** 


"The Hospital" Nursing Mirror.

Being the Extra Nursing Supplement of "The Hospital" Newspaper.

[Contributions for this Supplement should be addressed to the Editor, The Hospital, 428, Strand, London, W.C., and should have the word "Nursing" plainly written in left-hand top corner of the envelope.]

### News from the Nursing World.

### THE ROYAL WEDDING.

Our friends, the Pension Fund Nurses, favour us with numerous suggestions regarding the Royal wedding day. Some seem to have mixed up the words "presents" and "picnics," but it is on the latter subject only that we want their united opinions. Do they wish to join in a river picnic or a sea trip? and will they kindly decide, without delay, which of the two will give most general pleasure? Simple replies, given in as few words as possible, are all we require at present. Details of arrangement must come later on, after it has been distinctly proved that the members of the R.N.P.F. are unanimous in wishing to celebrate the occasion by an open-air *fête* of some kind.

### THE NURSE DOLLS.

Some of our nurse friends are sending their dolls in good time, and we beg to remind those whose dainty needlework is not yet finished that June 17th is the date fixed for receiving contributions to The Hospital Collection. All packages should be directed to the Editor, 428, Strand, and marked "Dolls" in left-hand corner of address card.

### UNIVERSITY COLLEGE HOSPITAL.

Again this year about seventy of the sisters and nurses of the above hospital enjoyed a day's outing at Richmond. On the 8th inst. the whole party drove down, half the nurses starting from London at eleven a.m., the remainder at four p.m. Luncheon, tea, and supper were provided at the Castle Rooms. Nearly all the nurses went on the river, a few only preferring a drive. Delma's Band played in the room at luncheon, and the Town Band on the lawn during tea. This annual treat is organised by Mr. Newton H. Nixon, the secretary of the hospital, and the expenses are met by private contributions from members of the Council of the College, of the Hospital Committee, Medical Committee, and others. We strongly commend this excellent plan, for ensuring a pleasant day's recreation for hospital nurses, to the secretaries of those institutions where a summer excursion is not in the annual programme.

### INTERESTING CASES.

Like many other popular expressions, the words, "interesting case," admit of various explanations. When used by a doctor, it is easy to follow his meaning and to see that it is for the advancement of science, and for the benefit of many future sufferers, that he points out the symptoms of the obscure or uncommon disease. But if a nurse, talking glibly of the cases in her ward, lays special emphasis on the "interesting ones," we listen with very different feelings. Is the so-called interest attached to the disease or to the patient? Generally to the former, we fear. Surely a nurse blunts the keen edge of kindly sympathy when she begins to think of suffering men, women, or children merely as "interesting cases." The expression, "a good case," has a far pleasanter sound, because it generally implies that the doctor and nurse have obtained a victory over their common enemy—Disease, and the science of the former and the skilled attendance of the latter have rescued some sick person from the very gates of death—truly "a good case," and one to be proudly rejoiced over.

### SICK ROOM VISITING.

It is impossible for anyone to do much private nursing without longing for the existence of some kind of universal code for the guidance of visitors to patients. Of course, such a thing is practically impossible, as independent views obtain in each household on the subject of invalids. *The Trained Nurse* for June contains a capital article by Mrs. Monson on "Etiquette of the Sick Room," in which the writer points out many things too often little thought of. She speaks of the "fervour of friends" passing off when the patient is convalescing, although at that stage the offerings of fruit, flowers, &c., would be of far more value than during the early days of acute illness. Remarks on the best position for a visitor to adopt in the sick room, the desirable length for calls, are very good. Mrs. Monson has evidently taken up the subject from every point of view, and our readers will cordially endorse her suggestion that "it is well to be a good listener in the sick room as in the social world," and also to avoid speaking of depressing news, unfortunate or sensational affairs, when admitted to the presence of anyone in weak health, either before or during the convalescent stage, which deserves so much more sympathy than is usually accorded it.

### SHE HAD COME TO STAY.

An old lady arrived at a small London hospital the other day, late in the afternoon, with a little bag in her hand, and was directed to the out-patient department. Here she met with civility and received advice, and was finally questioned as to her age and address. The latter was somewhere near Margate, and the old lady proudly declared herself to be nearly eighty years old. "It is a long way for you to go home to-night," said a sympathetic listener. "Oh, I'm come to stop," said the stranger firmly. To that she adhered, representations that her whole available capital of ten shillings would not pay her expenses for half a week, were in vain, and she was indifferent to the argument that only paying patients were received in the small institution. "It was surely a hospital," she said; "she needed treatment, and she would hand over her half sovereign willingly, but she was not going back." She had brought her bag, and she meant to stay. In the face of this weak old woman of eighty the staff felt completely powerless, and eventually they agreed that the self-elected in-patient should stay for the night at any rate. Happily the story got repeated outside, and a sympathiser promptly sent a little donation. So the old lady dried the tears which had flowed freely while her fate was undecided, and we trust that her health will be quite restored ere she sets out on her return journey to Margate!

### PROBATIONERS AT BRISTOL.

The annual distribution of prizes to the nurses at the Bristol General Hospital took place last week, Nurses Steen, Tait, and Fryer being the "second year" nurses singled out for distinction, and Nurses Bryan, Sampson, and Coke being the "first year" prize winners. Several pleasant speeches were made on the occasion, and in regretting the loss of the Matron, Miss Bann, who now holds a similar appoint ment at Liverpool, the hospital was congratulated on having secured so able a successor as Miss Morris. Dr. Solly's lectures, which preceded the examination, will long be remembered and valued by the nurses privileged to hear them. The nursing staff at the Bristol General Hospital includes about eighty members, thirty being on the private staff. Dr. Solly's departure is universally regretted, his services during his three years' residence having been greatly appreciated. The Matron, as well Dr. Solly, spoke very warmly of the satisfactory way in which the examination questions had been answered by all the candidates.

### BUSY BEES AT BARROW.

At Barrow-in-Furness there exists a children's association, formed under the auspices of *The Barrow News*, and called the "Busy Bee Society." The young folks belonging to it certainly merit their pleasant title, for they have been working very hard, and the "honey" they have collected has taken the form of a pretty child's cot, completely furnished, and accompanied by a handsome assortment of toys. Moreover, a useful bamboo screen was included amongst the offerings which the "Busy Bees" formally presented to the committee of the North Lonsdale Hospital, with an intimation that the young donors proposed to collect a sufficient sum annually, to defray the expenses of the occupants of the cot. A piano, purchased by subscriptions from a number of friends, was presented to the hospital on the same occasion, and is intended for the enlivenment of the patients, by whom music is greatly appreciated.

### REST FOR QUEEN'S NURSES.

The Galashiels Branch of the Q.V.J.I. has published its third annual report, which shows that the services of the nurse are highly appreciated and utilised by the sick poor in the district. It has been arranged for part of the Home to serve as "a Rest" for Nurses who may require a holiday or change of air after illness. Mrs. Archibald Cochrane has generously provided, for those who are not in a position to pay their own expenses, to have a free rest.

### WALES.

The Welsh Branch of the Queen Victoria Jubilee Institute continues to do good work, but it needs more funds. At Cardiff especially an appeal has been made for increased subscriptions. For three successive years a benevolent stranger gave £100 to the District Nursing Fund, and it is to be hoped that similar help will soon be forthcoming to secure equal assistance for the current year.

### LIBERALITY IN NORWAY.

The natural beauties of Norway are familiar to many of us, either from report or through personal experience, but its laws are probably little known to strangers in search of health or novelty. For instance, we learn from the *British Medical Journal* that each member of the Legislature is entitled to 13s. 4d. a day "while engaged in the service of his country, in addition to travelling expenses and allowances." Besides this, however, our readers will be interested to hear that these gentlemen have a right to free medical attendance during the session, and also, if they be ill, they get nursing massage, dentistry, wine, if ordered by a doctor, baths and gymnastics provided by the State. Truly, this forms a curious contrast to the distinction made in England between the classes that ought to pay and those who need not. The attempted supervision of hospital out-patient departments in England, and the endeavour to materially reduce the ranks of the claimants of advice and attendance, would hardly be understood by the Legislature of Norway.

### AMBULANCE SERVICE IN ITALY.

An interesting and picturesque scene is witnessed every Sunday at Rome. In the lovely grounds of the Villa Malta, on the Aventine Hill, the members of the Volunteer Ambulance Service assemble to practise the transporting of the sick—their removal on stretchers, &c. A suitable uniform is worn, and a white armlet adorned with the Maltese Cross, and also that of the Geneva Convention. The Association was formed by "the Knights of Malta" for aiding the sick and wounded in time of war. A hut hospital, with eighty beds, has been organised, with an admirably complete equipment for the patients, doctors, nurses, and servants. The "hospital trains" are three in number, and there is also a mountain train for conveying the sick on mule-back. By all of these, journeys for practice are frequently made, and during the last military manœuvres in North Italy the hut hospital proved of practical service.

### EUROPEANS IN INDIA.

Much has been said and written on the necessity for securing trained nursing for Europeans at up-country stations in India. The Queen has recently consented to become patron of the association which has been formed to meet the demand, and no doubt rapid progress will result from her interest. We trust the society will be fortunate in securing experienced trained nurses, for women unaccustomed to travel have but little idea of what is involved in it. Nurses who do well enough when their work is supervised, often turn out dismal failures when left to their own resources. There is little doubt that the Committee of the Up-Country Association will take every precaution, when the time of selection arrives, to send out a specially selected contingent of nurses to their fellow-countrymen and women in India.

### SHORT ITEMS.

A new municipal hospital is to be erected in the north of Berlin, making the fourth of these model institutions. It is proposed to have a department for the training of midwives and a nurse-training institute in connection with it.—The Chertsey Board of Guardians have decided to engage three new nurses, and to make a fresh departure as regards the nursing in the infirmary. One of the Guardians remarked at the meeting that they would never be able to keep really efficient nurses unless they made them more comfortable.—Dr. Hawkins has been appointed Hon. Treasurer to the Nurses' Co-operation, 8, New Cavendish Street, and Hon. Physician to the Nurses.

### Sanitation for Nurses.

By a Sanitary Inspector.

### X.—SOME RECENT SANITARY PROVISIONS.

(*Continued.*)

The law as regards the conveyance of any person suffering from a "dangerous" infectious disease (that is scarlet fever, diphtheria, typhus, typhoid, &c.) is most explicit, and as it is very important, we will quote the sections in full. "It shall not be lawful for any owner or driver of a public conveyance knowingly to convey, or for any other person knowingly to place in any public conveyance, a person suffering from any dangerous infectious disease, or for a person suffering from any such disease to enter any public conveyance, and if he does so he shall be liable to a fine not exceeding £10. And if any person so suffering is conveyed in any public conveyance, the owner or driver thereof, as soon as it comes to his knowledge, shall give notice to the sanitary authority, and shall cause such conveyance to be disinfected, and if he fails to do so, he shall be liable to a fine not exceeding £5, and the owner or driver of such conveyance shall be entitled to recover in a summary manner from the person so conveyed by him or from thepersons causing that person to be so conveyed, a sum sufficient to cover any loss and expense incurred by him in connection with such disinfection." It shall be the duty of the sanitary authority when so requested by the owner or driver of such public conveyance, "to provide for the disinfection of the same, and they may do so free of charge."

As regards a person who has died of a "dangerous" infectious disease, it must not be forgotten that the corpse can convey infection and hence the utmost care must be taken to prevent the spread of the disease amongst the inmates of the house. In some cases the district nurse will have a very hard task before her to persuade the friends to take the necessary precautions. Every hospital nurse has had experience of the unreasonableness of the friends, and in a private house, and without the restraints of a public place like a hospital, this difficulty will be greatly increased. The law says that "the body may not be retained unburied for more than forty-eight hours, elsewhere than in a room not used at the time as a dwelling place, sleeping place, or work-room, without the sanction in writing of the medical officer of health or of a legally qualified medical practitioner." A fine of £5 is imposed on any person who commits this offence.

Nor may the friends of a person who has died in a hospital of any dangerous infectious disease, remove the body except to convey it to a mortuary and with the distinct understanding that it shall be immediately buried, if the medical officer of health declares that it is necessary to do so for the public safety, and to prevent the spread of such infectious disease. Those who act contrary to this will incur a fine of £10.

Any person hiring or using a public conveyance other than a hearse, for conveying the body of a person who has died from any dangerous infectious disease, without previously notifying to the owner or driver of the conveyance that such person died from infectious disease; or if the owner does not, immediately after the conveyance has to his knowledge been used for conveying such body, provide for the disinfection of the conveyance, he shall, on the information of the sanitary authority, be liable for a fine not exceeding £5, and if the offence continues, for further fine not exceeding £2 for every day during which the offence continues. Several sections in the Act provide for the provision and maintenance of carriages suitable for the conveyance of persons suffering from any infectious disease, viz., ambulances, and pay the expense of conveying therein any person so suffering to a hospital or other place of destination. Such conveyances to be properly cleansed and disinfected, and such buildings and horses maintained and such persons employed, and all necessary things to be done as shall be considered proper for the purposes of such conveyance. Metropolitan Asylums' managers may allow any of the said carriages, with the necessary attendants, to be also used for the conveyance of persons suffering from a dangerous infectious disease, to and from hospitals, and places other than hospitals, provided by the managers, and may make a reasonable charge for that use.

The Metropolitan Asylums' managers shall continue to maintain the wharves, landing places, and approaches thereto heretofore provided of them, whether within or without London, and may use the same for the embarkation and landing of persons removed to or from a hospital belonging to the managers, and for any other purpose in relation thereto. The Metropolitan Asylums managers are required to provide on the banks of the Thames, wharves, or landing places beyond the metropolis, with convenient approaches thereto, respectively for the embarkation and landing of persons removed to or from any hospital ship or hospital belonging to the managers.

Regarding mortuaries, every sanitary authority is obliged to provide and fit up proper places for the reception of dead bodies before interment called a mortuary, and may make bye-laws with respect to the management and charges for the use of the same; they may also provide for the decent and economical interment at charges to be fixed by such bye laws of any dead body received into a mortuary. A body has to be removed into a mortuary under the following circumstances: Where either

(1) "The body of a person who has died of any infectious disease is retained in a room in which persons live or sleep;" or (2) "The body of a person who has died of any dangerous infectious disease is retained without the sanction of the medical officer of health or any legally-qualified medical practitioner for more than forty-eight hours, elsewhere than in a room not used at the time as a dwelling-place, sleeping-place, or work-room"; or (3) "Any dead body so retained in any house or room, so as to endanger the health of the inmates thereof, or of any adjoining or neighbouring house or building, a justice may, on a certi ficate signed by a medcal officer of health or other legally qualified medical practitioner . . . if it is the body of a person who has died of an infectious disease, or, if he considers immediate burial necessary, direct that the body be buried immediately without removal to the mortuary."

It is important to notice that under head (1) "any infectious disease" is mentioned instead of the words "dangerous infectious disease." This greatly extends the powers of the medical officer of health, as it authorises him to have the body removed to a mortuary when death has resulted from any one of the infectious diseases not scheduled, if he considers such removal necessary. Likewise, under head (3), a justice may give an order for removal in case of death from "an infectious disease," here the limiting word "dangerous" being again omitted.

It is very important that these regulations regarding infectious diseases, whether "dangerous" or otherwise, should be very thoroughly understood by all nurses, and hence, even at the risk of being wearisome, we have made a point in many instances of actually quoting the exact words of the Act, so that there should be no chance of misunderstanding.

### Wants and Workers.

"District Nurse" will be grateful to anyone who will give plain needlework or dressmaking to a lady in reduced circumstances.

Will Mrs. Franklin, late Matron to the Paignton Hospital, forward her address to Nurse O., Adelaide House, Great Malvern?

Could anyone recommend me a suitable place (a home or otherwise) to send a paralytic young man to for a summer holiday? Seaside preferred. Terms must be moderate.—*Maida Vale.*

### Nursing in India.

(By Our Indian Correspondent.)

### THE INDIAN NURSING SERVICE.

Outsiders and military people who may have the welfare of our sick soldiers at heart, cannot view the many changes in the Indian nursing service with anything but sorrow. There are still eight vacancies, and as no less than 11 new Sisters were appointed last trooping season, it shows that in a very short period 19 have departed. This is a large number out of a service whose full complement only reaches 52. During the last 12 months one Sister has died, and a few have left to be married.

Some months ago I noticed in the papers that the re-engagement for another term of five years was gazetted of Miss Otley, Lady Superintendent, Madras, and Miss Beresford, Deputy Superintendent, Rangoon. Since then I have observed, their resignation and withdrawal from the service. Some of the Nursing Sisters in the same Presidency have also resigned.

This is probably owing a good deal to the unsatisfactory management of the service, duties not being sufficiently defined, and where the medical officers are not favourably disposed towards the ladies of the nursing staff their usefulness is much handicapped.

The appointment of Miss Maxwell Müller as Lady Superintendent of Madras has given general satisfaction. She was first on the list, and her wise government, and talents as a nurse, have made her respected throughout the Presidency.

The Lady Superintendents of the nursing service furnish confidential reports once a year on the nursing staff in their domains. These are supposed to be submitted to the medical officers of the district in which the nurses work.

"The Lady Superintendent will once a year visit the female nursing staff of all hospitals in her circle, and will afterwards submit to the Principal Medical Officer of Her Majesty's Forces, through the Medical Officer in charge and the Administrative Medical Officer of the district in which the lady nurses are employed, for such remarks as these officers have to offer, a confidential report (on Army Hospital Form No. 60) on the conduct, capabilities, and manner in which each Acting Superintendent and Nursing Sister has performed her duties. In the case of a Nursing Sister, the Lady Superintendent will also state whether she is fit in every respect for advancement to the post of Acting Superintendent."

Some of these ladies, however, think that they require no assistance from the medical side at all, and on their own judgment alone send up reports to headquarters. The opinions of medical officers, who are daily in contact with the nurses and their work, are therefore completely ignored.

While this tour of inspection goes on, the ladies receive five rupees extra per diem for detention, and have their travelling expenses paid.

One of the last things Lord Roberts did as regarding the nursing service, was to recommend to the Government of India that the sphere of employment of the Indian nursing service might be extended to the supervision of the nursing arrangements of the station hospitals for British soldiers' wives and children. The recommendation has been approved of, and is to be carried out.

The latest appointments in the service are: Miss James, officiating Lady Superintendent, Eastern Circle, Rawal Pindi; Miss Moore, officiating Lady Superintendent, Western Circle, Meerut; Miss Murray, officiating Lady Superintendent, Madras Circle, Bangalore; Nursing Sister Still as officiating Deputy Superintendent, Mian Mir; Nursing Sister Bolster as officiating Deputy Superintendent, Quetta; Nursing Sister Hayes as officiating Deputy Superintendent, Peshawar; Nursing Sister Pearse as officiating Deputy Superintendent, Lucknow; Nursing Sister Kelly as officiating Deputy Superintendent, Bareilly.

The transfers are: Miss Hislop, Deputy Superintendent, Allahabad; and Miss Wildman, Deputy Superintendent, to Umballa.

The promotions are: Miss Maxwell Müller, Deputy Superintendent, to Lady Superintendent; Nursing Sister Stainstreet to Deputy Superintendent; Nursing Sister Stokey to Deputy Superintendent; Nursing Sister Jardine to Deputy Superintendent; Nursing Sister Murray to Deputy Superintendent.

### Queen Victoria Jubilee Institute for Nurses.—Rural Branch.

The annual meeting of the Rural District Branch of the Queen Victoria's Jubilee Institute for Nurses, took place at Grosvenor House on the 7th inst. The Duke of Westminster presided, and read several letters of apology from friends unable to attend. The room was fairly filled, and much interest in the object of the meeting was shown by all present.

The Duke of Westminster explained that about sixty districts are already supplied with Queen's nurses, but a great many more being needed, it is necessary that funds should be forthcoming for training an increased number of women. He said that a friend of his had promised £100 per annum, and he could not resist following so good an example, and he hoped others would feel the same. Although landowners were suffering so much from the prevailing depression, he trusted there were still some who were not quite ruined, and who would acknowledge the claims of this society. He spoke of the Q.V.J.I. having in no wise snubbed this association; on the contrary, it had encouraged and helped it, so far as means permitted.

Lord Halsbury, speaking next, said that the Duke had left him very little to say, for he had well explained the objects of the association. He himself thought that many sick people needed good nursing more than they did medical treatment, and he trusted that he should not get into trouble with any doctors who were present, by this remark. Whatever differences of opinion might exist as to the diffusion of so-called charity in other quarters, there could be no objections raised against benevolence flowing in this channel. More nurses were needed, and evidently money must be found to train them for the work.

Mr. Martin gave many interesting details of the plan now followed by the Rural Branch, which had been previously found to work successfully in his part of the world. During twenty years he had had experience of nurses of the cottager class, and, recently, of cultured women. He was quite sure poor people made the latter more welcome, and found them more useful, than they did the former. The ladies could teach them simple hygiene and sanitation, and their example proved most valuable in dealing with disinfection, &c. These qualified nurses also instructed the friends in invalid cooking and in attending intelligently to the patient during their own absence. In securing an inspector they had done wisely, for even the best nurses needed keeping up to the mark. He regretted the absence of Sir Henry Acland, who had sent them an admirable letter showing his interest in, and knowledge of, the subject.

Lady Victoria Lambton gave an excellent account of the progress made in district nursing, speaking of the old order of things, and the difficulties formerly experienced in getting suitable persons to nurse the sick poor. Of the many applicants for the post, she always found those who had no training whatever generally considered themselves vastly superior to those who had some experience. She explained very lucidly the system on which district nursing was managed, the rates of payment made by patients, &c. The benefit it was to the doctors to know that their orders would be intelligently carried out, whereas formerly, the ignorance of those who nominally "looked after" the poor, made proper treatment obviously impossible. The terrible suffering of young mothers and the thousands of avoidable deaths annually taking place were admirably detailed by her ladyship, who said that most of them resulted from "the audacity of ignorance." She hoped the day would come when a Home of Rest and Pensions for broken down nurses would be within the means of the Rural Branch. Whilst people could find money to support hospitals for horses, dogs, and even pet birds, surely we had a right to expect subscriptions to a fund for supplying trained nurses to the suffering poor. The appeal made so eloquently by the Duke of Westminster and other speakers ought certainly to result in largely increased donations and subscriptions to a most practical and admirably managed association.

### Presentation to Mrs. Garrett-Anderson.

At the annual meeting of the Association of Registered Medical Women held at the New Hospital on June 6th, a presentation of plate was made to Mrs. Garrett-Anderson, M.D., on the occasion of her resignation of the post of visiting physician to the institution.

Mrs. Marshall, M.D., first read an address, expressing in the most cordial terms the great regret of Mrs. Anderson's colleagues on the staff at losing her from the active work of the hospital.

Dr. Elizabeth Blackwell, on behalf of seventy British medical women, presented Mrs. Anderson with a very handsome set of candelabra and a loving-cup, as tokens of the great regard in which she was held, and as an acknowledgment of all they owed to her, both as their pioneer in England and as the founder of the New Hospital. Mrs. Anderson, in accepting the testimonial from her colleagues, referred to the fact that Dr. Elizabeth Blackwell had done in America all that she herself had been happy enough to succeed in doing in England, and that it was the highest reward to Dr. Blackwell and to herself to see women prospering in the medical profession, and gaining the confidence and respect of the profession and the public. The road into the profession was now fairly open, both in England and America, though in both countries women had still a good many things to contend with if they aimed at a high degree of knowledge, or of a large measure of success as practitioners. She hoped, however, that in a few years all hindrances would be removed, and that women would be encouraged and helped to do their best, whatever that might prove to be.

In accepting the beautiful gifts now offered her, she could not but think that the seventy kind donors had been very happily inspired in their choice. They were giving her candlesticks and a loving cup, symbols of light and fellowship. Could they have given her, or could they desire for themselves anything better?

In connection with the New Hospital for Women in Euston Road there is a delightful convalescent home at Aldbury, near Tring, where patients are sent to recover their strength. It is only a cottage, but a very pleasant one, and has been suitably furnished by various friends There is accommodation for five patients only, and the arrangements are essentially home-like. The place has been open about a year, and on Saturday last an At Home was given to the medical women and others interested, and a pleasant afternoon was spent in this charming neighbourhood.

### London Skin Hospital, Fitzroy Square.

### ANNUAL MEETING.

The London Skin Hospital, Fitzroy Square, of which H.R.H. the Duke of York is patron, held its sixth annual meeting on the 8th inst. In the absence of the President, the Marquis of Carmarthen, who was unavoidably detained at a Private Bill Committee, the chair was taken by Mr. Charles Pritchard. Mr. and Mrs. Startin, Mr. Sharbord, Dr. Shuldham, Dr. Sanctuary, Mr. Barnes Nowell, Mr. Henry Baker, and other ladies and gentlemen were present, and letters of regret were read from Canon Barker, Mr. Pindar Simpson, Mr. Lion, and Mr. H. C. Ash. Lord Carmarthen was re-elected President, and the Dukes of Northumberland and Argyle and the Earl of Rosslyn, Vice-Presidents. The report stated that the out-patient and bath departments were working satisfactorily, and the additional accommodation for in-patients was certainly most convenient, but the expense incurred in carrying on the latter was considerable. A competent Nurse-Matron had been secured, and it was most desirable that a supplementary fund should be raised to help those persons whose means were insufficient to pay the full fees. It was constantly necessary for the doctors to decline the applications of people whom they knew urgently required treatment because they could not meet the expense. It was, therefore, decided to organise a supplementary fund for this purpose. Donors of certain amounts are entitled to send patients into the hospital for longer or shorter periods, according to the sums subscribed. The Committee trust that the list of subscribers will be largely increased during the current year.

### Appointments.

Carmarthenshire Infirmary.—Miss Annie Harries has been appointed Matron of Carmarthenshire Infirmary. She was trained at St. George's Hospital, and afterwards held the post of Sister there. Miss Harries has had considerable experience in private nursing and also in housekeeping, and we wish her every success.

### Minor Appointments.

Bristol General Hospital.—Miss Hayes has been elected Night Superintendent at this institution. She was trained at the General Hospital, Birmingham, and held the post of Sister of one of the medical wards there.

Bristol General Hospital.—Miss Hill, late Night Sister, has been appointed Assistant Matron at the Bristol General Hospital.

Wolverhampton and Staffordshire General Infirmary.—Miss Hepper had been appointed Night Superintendent of this infirmary. She was trained at the West London Hospital, and has since had charge of a ward in the Cumberland Infirmary. She has excellent testimonials, and we wish her success in her new work.

Naval Hospital, Plymouth.—Miss Florence Moore has been appointed Sister to the Naval Hospital, Plymouth. She was trained at St. Bartholomew's Hospital, and for the past twelve months has been Night Superintendent at the Royal United Hospital, Bath, from which she takes with her everyone's good wishes.

Pembrokeshire and Haverfordwest Infirmary.—Nurse Spratt has been appointed Sister at this hospital. Nurse Spratt has has worked for five years as assistant and staff nurse, and her services are greatly valued in the infirmary.

### MEMORIAL TO THE LATE MRS. WARDROPER.

A tablet to the memory of Mrs. Wardroper, the late Matron, is to be placed in the chapel of St. Thomas's Hospital. A clay model of the design suggested was submitted to the General Court of Governors on the 14th inst.

### Medico-Psychological Association.

EXAMINATION FOR NURSING CERTIFICATE, MAY, 1893.

1. Mention the causes of lung disease.
2. By what means (*i.e.*, by what channels is, the refuse or waste matter of the body drained from the circulation?
3. What symptoms would lead you to expect that a patient is losing weight?
4. What symptoms would lead you to expect that a patient is gaining weight?
5. (*a*) What is a sensory nerve? (*b*) What is a motor nerve?
6. Name the special senses.
7. (*a*) What is a drawsheet? (*b*) Explain how you would use it? (*c*) What are its advantages?
8. (*a*) What observations would you make regarding the passing of urine, and (*b*) The appearances of the urine?
9. (*a*) Why is occupation important in the treatment of the insane? (*b*) What rules should be observed in promoting the occupation of patients?
10. (*a*) What patients are most likely to escape? (*b*) What circumstances would make you suspicious? (*c*) How would you guard against escape?
11. (*a*) In what way should attendants conduct themselves towards patients? (*b*) What do you understand by "showing a good example"?
12. (*a*) What are the risks in treating cases in private houses compared with asylums? What precautions would you take? (*b*) What are the difficulties with relatives in private houses, and how would you endeavour to meet them?

The following are the names of the candidates who passed the examination held on May 1st, 1893:—

Derby Borough Asylum.—Nurses: Ida Adam, Grace E. Gomm, Annie Morris, Mary A. Doughty. Attendants: John A. Froggatt, Henry Bodkin, Charles Flixon, William T. Dring.

Wadsley Asylum, Sheffield.—Nurses: Kate E. Wheatley, Elizabeth Turner, Mary J. Wright, Zillah Barwell, L. Cash. Attendants: George Thompson, Thomas E. Addey, Alfred Whitehead, Alfred H. McCreadie, William Boag, Thomas O'Dowd, S. Greenwood, S. H. Tonks, Ernest. A. Byrnand, Gerald Cricker, H. Coopland, Henry Sunley, Fred Wood.

Royal Edinburgh Asylum.—Catherine McKeith, Elizabeth Burgess, Annie Byfield, Janet B. Currie, Margaret Johnstone, Isabella McNab, Isabella Smith, Isabella Mc W. Shaw, Jane Wood, Jane Macdonald, John Stove, John Petrie, and James Mitchell.

Kirklands Asylum, Bothwell.—Agnes Craig, Florence J. Hooper, Mary Johnston.

Larbert Asylum, Sterling.—Robert Boyd, Donald Hendry, Alexander McCorquodale, John Macdonald, Samuel Thomson, Margaret Clark, Mary Jane McNair.

Melrose (Roxburgh) District Asylum.—William Leith, Helen Craise, Margaret Stevenson, Jane Smith.

Fife and Kinross Asylum.—Helen Buchan, Jessie Mitchell, Maggie MacLean, Ada Isabel Taylor, Janet Wallace.

Argyll and Bute Asylum.—Neil Livingston, Collin Mitchell, Peter Mitchell, William Ramsay, Marianne Brown, Maude Barnaby, Agnes Beaton, Ann Lochead, Margaret McLeod, Christina McCulloch, Mary Gillies.

Brookwood Asylum, Surrey.—Charles Carpenter, James Thomas Harding, James Peto, Walter Sutton, Charles Tompson, Alfred Wilkinson, Mary Donohoe, Clara Lyons, Ada Luckhurst, Mary Penna, Mary Rea, and Mary Ann Stevens.

City of London Asylum, Stone.—Samuel Haylock, Albenia Cooper, Mary Driver, Emma Jarvis, Charlotte Mertling, and Mary Thomson.

Derby Boro' Asylum.—Henry Bodkin, William T. Dring, Charles Flixon, John Arthur Froggatt, Ida Adam, Mary Ann Doughty, Grace E. Gomm, and Annie Morris.

Bethnall House Asylum, London, E.—Clara de Pradines, and Mary Heynes.

Rainhill Asylum, Lancashire.—Hugh Allen, William R. Colwill, Alfred John Fluck, Hugh H. Howard, James Howitt, William Riley, Frank Smith, Frederick Charles Scriven, Edward Thomas, George Wait, John Samuel Wakeling, Agnes Gregson, Emma Lyle, Annie Sellar, and Mary Weller.

Holloway Sanatorium.—Alfred Bishop, William Blades, William Montague, Ellen Cox, Frances Davis, Emma Daniel, Rose Heath, B. Topham Jones, Katherine Elizabeth Johnson, and Mary Roylance.

Royal Asylum, Dundee.—Francis Dick, William Mitchell, James Nicholl, John Simpson, James Stewart, William Walker, and Mary Swanson.

Warneford Asylum, Oxford.—Frederick John Swadling, Sarah Warland, and Emily Prior.

Winson Green Asylum, Birmingham.—John Banford, William Crawford, Edwin Thomas Jones, Charles Henry Underhill, Julia Berks, Hannah Coles, Emily Cullam, Emma Moore, Kate Emily Keen.

West Riding Asylum, Wakefield.—Harriett, Allen, Lillian Backhouse, Alice Dearnley, Alice Lindsay, Nellie Megson, Maria Penty, Emily Mary Talbot.

West Riding Asylum, Wadsley, Sheffield.—Thomas Ellis Addy, William Boag, Ernest Byenand, Harry Coopland, Gerald McCaul Crickmer, Samuel Greenwood, Alfred Thomas McCreadie, Thomas O Dowd, Harry Sunley, George Thompson, Samuel Hale Tonks. Alfred Whitehead, Frederick Wood, Zillah Barwell, Lizzie Cash, Elizabeth Turner, Mary Jane Wright, Kate Elizabeth Wheatley.

West Riding Asylum, Menston, Leeds.—John William Bennett, Henry Hearn, Charles Henry Illingworth, John Myers, George Yerry, John William Watkinson, Esther Ayres, Constance Kate Gates, Louisa Hampshire, Sallie Jones, Clara Plummer, Annie Mitchell, Kate Viney.

### On the Changing of Probationers.

By a Ward Sister.

An excellent address to Queen's probationers appears in the June number of *Nursing Notes*. It is written by Miss Mary Dunn, Superintendent of the Irish Branch of the Q.V.J.I.N., and the principles it inculcates apply equally to all probationers in nurse training schools.

Perhaps each set of hospital rules does not verbally include the injunction to render ready and cheerful obedience to all the rules of the institute, but the spirit of this regulation is universally dictated. The feeling of opposition indulged in by probationers is a far more serious fault than they have any conception of. When they are comfortably settled in a ward they look upon it as an almost permanent arrangement, considering any change a "grievous calamity." Even Queen's Nurses, who are trained and wiser women generally than inexperienced probationers, seem to hate changes. As Miss Dunn says to them: "I have seldom heard of nurses who are glad to leave the central homes, and in one way this is a good thing. It speaks well for the homes, well for your superintendents, and well for your fellow workers; but like other good things this natural and right feeling may be carried too far if it makes you unwilling to obey the call of duty when asked to work in other spheres."

This is equally true of wards, and a popular sister knows exactly the expression which will disfigure the pleasant face of a probationer when she tells her that her services are required elsewhere. And, moreover, it is a little unfair to the new work for it to be taken up in a discontented spirit. The transferred probationer fails to see that she is guilty of injustice to the fresh sister and her fellow-workers in going grudgingly to their aid. Possibly young nurses do not realise how depressing it is to busy people to have needless friction introduced.

Perhaps a few days after her first appearance in a ward a girl will say cheerfully, "To tell you the truth, Sister, I did not want to be moved, but I find I am just as happy in your wards after all." Some such light speech as this convinces the speaker that she has quite cancelled the effects of her previous ungraciousness, forgetting that no person can indulge in even temporary discontent without insensibly affecting those with whom she is in the close contact demanded by ward duties.

Another of Miss Dunn's telling paragraphs ought to appeal strongly to the conscience of every probationer who has taken up nursing in the right spirit, "Those who signed agreements are bound to go, but I hate the idea of your having to be forced out in this spirit. What would be your opinion of soldiers who enlisted on the understanding that they would remain soldiers only so long as there was no fighting to be done; or that, when that did come about, their officers had to drive them into the field at the bayonet's point, you would scarcely consider them fit recipients of badges, medals, certificates, or such like, would you? Remember that

"'Not once nor twice in our rough island story
The path of duty was the way to glory.'"

### Trained Nurses in Workhouses.

### SHEFFIELD UNION INFIRMARY.

The humanity and general advantages of efficiently nursing the sick poor has been fully recognised in this Union during the last three or four years, and has been attended with every success. The hospital wards, under the able superintendence of Miss Thomlinson, are now entirely nursed by a carefully selected staff of fully trained and certificated charge nurses, whilst a number of probationers on the three years system are steadily growing up to efficiency. The first six of these to complete the full term of training have recently been subjected to an oral, written, and practical examination by an outside examiner. It is pleasing to note his report, which was ordered to be printed on the minutes of the proceedings of the Guardians, and is as follows:—

I have the honour to report that the following nurses have been successful in the recent examination held by me at your request at Fir Vale. Their answering was most creditable to themselves and to their instructors. Their bandaging also deserves a word of praise; it was firm and neat, and I have no doubt would be effective in *bonâ fide* cases. In filling up their certificates I wish the words "Very good" to be inserted in the appropriate places, both as to medical and surgical qualifications.

(Signed) John W. Martin, M.D., M.R.C.S.

Names of the Nurses: Ethel M. Mullett, Mary Black, Maud Hill, Margaret Browne, Jane Jones, and Charlotte E. Gardner.

### Where to Go.

Mr. Francis James is showing a charming collection of water-colour sketches at the Dutch Gallery, Brook Street, Hanover Square. The artist has brought back some delightful souvenirs of his late sojourn in Venice, and these will be very interesting to lovers of the beautiful water city. Many of the impressions he has succeeded in pourtraying are very characteristic of the varied moods and aspects of Venetian scenes. Mr. James shows us very few specimens of his wonderful flower painting on the present occasion, though those that are on view prove that his hand has lost none of its cunning in this direction.

Messrs. Cassell and Co. are holding their eleventh annual exhibition of original drawings in black and white at Cutlers' Hall, Warwick Lane, Newgate Street. The exhibition is open from ten to five until June 29th.

A delightful concert was given at St. James's Hall last week, in aid of the building fund of the Dental Hospital of London. The following artistes kindly gave their services: Madame Newling, Miss Margaret Hoare, Miss Clara Butt, Mr. Ben Davies, Mr. Watkin Mills, Mr. Roger Peek, Mr. Norman Salmond, Miss Frida Scotta, M. Joseph Hallman, and Madame Frickenhaus, and songs were contributed by the Guildhall Choir, under the direction of Sir Josepn Barnby, Mrs. Wilhelm Ganz being the accompanist. The Hall was well filled, and the audience enthusiastic. We hope the pecuniary result may be in proportion to the pleasure which all present derived from the music.

### For Reading to the Sick.

### SUFFERING WHICH LEADS TO GLORY.

The desire of glory is one of the most powerful of the agents which move the actions of men. The heroes of history were made by it, and a story is told which illustrates this truth. After the battle of Marathon, in which Miltiades, a Greek general, highly distinguished himself, Themistocles, who was then quite young, was observed by his friends to be very sad and unhappy, lying awake whole nights together, and wandering gloomy and alone by day, avoiding speech with every one. He was at length asked what ailed him, and replied, "The triumphs of Miltiades will not suffer me to sleep." He envied the general so much that he determined to gain greater glories for himself, and in a few years became one of the foremost men of Greece. He was successful because he had set his mind upon it, and was determined to have it, cost him what it might of trouble and sorrow and discomfort. We have often heard of people who have laboured and studied all their lives to get honour, and, indeed, have known many such. To be called the finest orator, or the first lawyer or actor of the day, to be fêted and extolled as the most famous statesman, to be written and spoken of as the greatest inventor, is sufficient reward for hundreds. Some are so foolish as to "spurn delights and live laborious days," merely to amass a huge fortune, whether by fair means or foul; they do not care so that the fame of their millions resounds throughout the world. All these work for the praise of men, and are bitterly disappointed when they do not receive it.

Why should not we work for a worthier end, and have our eyes fixed on the glory of heaven? If we set our hearts on it and determine to gain it, we shall press forward to obtain our desire, instead of groaning and complaining when we have to suffer in this world. Through suffering to glory was the path our dear Lord Jesus trod, and we should use energy, zeal, patience, prayer, for all are necessary to secure the prize—the glorious crown laid up for us above; whereas our energy flags, our zeal grows cold, our patience fails, our prayers are half-hearted, because we do not keep our end in view. And yet it is a lovely picture for the eye of faith to contemplate. "Sorrow vanquished, labour ended," no more trouble, no more evil, for death will be swallowed up in victory. When we have been victorious in the battle of life, we shall say with St. Paul, "I reckon the sufferings of this present time are not worthy to be compared with the glory that shall be hereafter."

### Notes and Queries.

### SPECIAL NOTICE.

The contents of the Editor's Letter-box have now reached such unwieldy proportions that it has become necessary to establish a hard and fast rule regarding Answers to Correspondents. In future, all questions requiring replies will continue to be answered in this column without any fee. If an answer is required by letter, a fee of half-a-crown must be enclosed with the note containing the enquiry. We are always pleased to help our numerous correspondents to the fullest extent, and we can trust them to sympathise in the overwhelming amount of writing which makes the new rules a necessity.

### Queries.

(149) *Monthly Nurse.*—Can you tell me of any hospital or institution where trained nurses can learn monthly work free of charge?—*A. F. W.*

(150) *Massage.*—Who is the best instructor in massage, and how long does it take to gain a thorough knowledge of it?—*Dora.*

(151) *Patient.*—What is the best way to get a resident patient into a doctor's house?—*D.*

### Answers.

(149). *Monthly Nurse* (*A. F. W.*).—We should advise your writing to the Hon. Secretary of the Workhouse Nursing Association, 6, Adam Street, Adelphi, London. The Hon. Secretary of the Trained Nurses' Club, 12, Buckingham Street, Strand, is also very kind in giving advice.

(150) *Massage* (*Dora*).—You will find the information you require in "Burdett's Hospital Annual," under the heading "Nurses and Nursing."

(151) *Patient* (*D*).—By advertising yourself or by answering advertisements, unless you are personally acquainted with any doctors, who would advise you.

### The Muses' Looking Glass.

In this very curious and interesting work* Mr. Leland has brought together a vast amount of material tending to show that in certain parts of Italy, and notably in La Romagna Toscana, "stregeria," or witchcraft, known as the old religion, has survived from early Etruscan days under the crust of Christianity which nominally superseded it. The difficulties experienced in gathering evidence about the ' folletti" or minor spirits, and in collecting details of magical cures, incantations and spells, seem to have arisen partly from the secrecy which naturally shrouds such observances, and partly from the fact that with the progress of modern education the number of the initiated is steadily decreasing. Mr. Leland was successful in gaining the confidence of "those who know," but from the hints which he gives in his introduction it would appear that the task led him through devious paths and among strange company. The first part of the book is taken up by an investigation into the survival of Etruscan deities under slightly altered forms or names. The spirit of Zinio or Tigna, the deity of thunder and lightning, is identified with Jupiter. Téramó is still invoked by thieves and merchants, and is obviously connected with Mercury. "Aplu is a spirit who greatly loves hunters; he is the most beautiful of all the male spirits. He is also a spirit of music." Here we have Apollo with scarcely a variation. The list of these "folletti" is a long one. The belief in them appears to be handed down in certain families and to exist invariably side by side, as a kind of second string to the bow, with the orthodox observances of the Church. Indeed it would seem that the priests not infrequently encourage the old forms of superstition in others or are themselves deeply tainted by them. A curious instance of this may be quoted.

"It seems to me strange," I remarked one day, "that no *men* seem to practise witchcraft." "Oh, but there are, though," remarked my head collectress. "Why, there is a priest here in Florence who is a 'streghone.' " "Santo, now if you had told me there was a thief in the police I should not have been astonished. But he can't be a real wizard." "Ma si, Gesualda there knows him. And you can see him yourself if you want to." I thought on the whole I did not want to . . . and asked how he came to practise our noble profession. "Ah," cried the witch with a smile, "he couldn't help himself; he had to become one. He was called in to confess a witch who was dying, and did not know with whom he had to deal. So she got her confession and then said she had something to leave him. Would he have it? Oh, wouldn't he! Si sicuro. 'Then,' she cried, 'I leave you my witchcraft.' And before he could say a word she was dead and off, and he found himself a wizard.' It appeared from another informant that the legacy came to the unhappy priest in the form of a casket in which he found a mouse. "And so the spirit of witchcraft came on him. And when it comes if the witch touches any person, he or she will be bewitched, and waste away or die. But this priest, being a good man, would not touch or embrace people at such times, but going into the country touched trees, or grain, or maize, and whatever he touched dried up. So he did as little harm as was possible; but for all this he could not help being a wizard." The remarkable part of the story is, as Mr. Leland points out, "that in the city of Florence, in the month of January, 1891, there were people who believe in a prehistoric Shahmanism, which is stronger and mightier than that of the Church." Later on the author discovered there was no great anomaly in a priest being a wizard, "for witchcraft is a business like any other. Or it may come upon you like love, or a cold, or a profession, and you must bear it till you can give it or your practice to somebody else." There is no devil in it, and its possession does not necessarily imply sin. In fact, "in some strange way, the 'strega' works clear of theology."

* "Etruscan Roman Remains." By C. G. Leland (T. Fisher Unwin.)

The practical outcome of all this is the survival among the modern Italian peasants of occult remedies depending on spells and amulets which have been handed down among them for over two thousand years. Marcellus Burdigalensis, a court physician of the fourth century, made a collection of one hundred magical cures employed in his days among the rural classes. Of these a large proportion are identified by Mr. Leland as existing at the present time. Sometimes the spells remain practically the same, allowing for the variations of language. Sometimes they have been partially Christianised by the substitution of some saint's name for that of the spirit originally invoked. In nearly all cases the remedies are purely magical—that is, the cure is not in any way dependent upon the natural properties of the herb or other substance employed.

A few examples will serve to show the intimate connection between the ancient and modern forms of superstition. Marcellus says: "If grass or ivy grows on the head of any statue, and it be gathered and tied in a red cloth to the head or temples, it will be a marvellous remedy for headache or neuralgia." The Florentine prescription is much the same in the present day. "When you take grass from the head of a statue to cure a headache, you must say—

'I do not take the grass or ivy,
But I take the magic power,
That the headache may leave me,
And may the devil carry away
The one who gave it to me.'

And then you must make 'le corne' (the sign of the horns or jettatura) behind you."

The spell of the hare is even more accurately preserved. Among the cures of Marcellus is the following for the colic: "Take from a live hare the ankle bone, remove the hair from his belly, and let him go alive. From that hair or fur make a thread, and with it bind the bone to the body of the sufferer, and you will see a wonderful cure. And when you shall have shorn away the fur, let the hare run away alive, and say—

'Run, run, little hare,
And carry the colic with you!' "

The process in modern Italian magic as revealed to the author is almost identical. An invocation is used in bringing the hare home, the fur is cut off in the form of a cross, and the animal is dismissed with the injunction,

"Go, and may'st thou bear
All the trouble and ill with thee;
And leave us free with good health."

But in addition, "Then spit behind you thrice, and look not behind you, and go not out from the house for three-quarters of an hour." Mr. Leland accounts for the more amplified form of the modern magic by the suggestion that Marcellus abbreviated many of his cures. It would seem that all the remedies are imperfect unless accompanied by the proper form of incantation, and it was the collection of these which proved the most arduous part of the whole undertaking. Some of these spells are purely pretty and innocent. For instance, the cure for sore eyes, which consists in the following verse, repeated at a well or fountain after seeing the first swallow:—

"I ran to where the fountain flies,
And in its water washed my eyes,
Which were so long my pain and grief,
Yet no physician brought relief;
But the first swallow which I see
Has caused a happy cure to me.
Blest may the swallows ever be."

### The Book World for Women and Nurses.

[We invite Correspondence, Criticism, Enquiries, and Notes on Books likely to interest Women and Nurses. Address, Editor, The Hospital (Nurses' Book World), 428, Strand, W.C.]

Further Recollections of a Happy Life. From the Journals of Marianne North. (Macmillan and Co.)

The many readers who delighted in the first volumes of Miss North's autobiography will welcome these additional chapters giving some account of her first European journeys, and one through Egypt and Syria. The ground traversed is more familiar than that of the wanderings in the earlier books, but there is none of the guide-book flavour which pervades so many books of travel. The writer often indeed seems almost afraid of imparting information in her adherence to personal impression and experience, but the result is a volume which will be read and enjoyed, while painstaking attempts at supplementing Baedeker are laid on one side. There is a rapid tour through Italy—"Milan, Verona, Brescia, Venice, who can describe them?" says the Journal. We wish she had paused to try. Florence and Rome are disposed of in a page "one is kept in a perfect fever of excitement"; she was disappointed with St. Peter's, worried at the accumulation of art treasures, and enjoyed most wandering in the gardens with her father. She seems to have shared his dislike to towns, and is happiest when they are away together in regions remote from civilisation. They passed through Turkey and across to Smyrna where the guard (a native of Norfolk) insisted on their travelling first-class, though they had taken third-class tickets in order to study the natives. "That don't matter, sir, I ain't agoing to allow them young ladies to go into third-class carriages which is only fit for Turks and sich; there's no saying what they mightn't catch." There was a hasty visit to Athens, where the Journal is again a little disappointing in its want of detail, and then home via Marseilles to Hastings. The following tour to Egypt and the Holy Land is by far the most interesting. The voyage down the Nile is described with great spirit; she had the gift of making herself readily at home among all the strange races with whom her wanderings brought her into contract, and her friends were of every variety, from the "little slippery black babies, which always reminded me of small black pigs, so round and pretty were they," and one of whom she nearly bought one day for two empty reels of cotton and a button, to the old Pilot, whom she doctored with lumps of camphorated sugar. This Pilot's description of her, imparted to a mutual friend, gives a pretty picture of her doings on the Nile: "This Bint was unlike most other English Bints, being, firstly, white and lively; secondly, she was gracious in her manner, and of kind disposition; thirdly, she attended continually to her father, whose days went in rejoicing that he had such a Bint; fourthly, she represented all things on paper, she drew all the temples of Nubia, all the sakkiahs, all the men and women, and nearly all the palm trees; she was a valuable and remarkable Bint!" They crossed from Alexandria to Jaffa, and so on to Jerusalem. As at Rome, she is disappointed and disillusioned, and glad to get away and up the steep slopes of the Lake of Gennesareth, "breathing the purest air and perhaps seeing one of the most glorious views in the whole world." They spent

Whit-Sunday under the cedars of Lebanon, and wandered freely in the wild and half deserted country, rejoicing in freedom from tourist association, and shunning as far as possible the beaten track. It is all as unlike as possible the stereotyped traveller's record. The associations of history and romance are all left on one side, or perhaps taken for granted. It is the actual present life of the people, the humours of the road, and transient, quickly-seized glimpses of the beautiful and picturesque which serve to make up those treasures of recollection which she tells us served to brighten so many odd hours on long journeys or in sleepless nights. Related in her flowing, easy style, with all the vividness of immediate record, they may well serve the same purpose for many stay-at-home readers who complain of dull hours and *ennui.*

---

The Cruise of the Wild Duck and Other Tales. From the Danish. (T. Fisher Unwin, Pseudonym Library.)

These short stories selected, as the preface informs us, from the writings of Holger Drachmann, are interesting from more than one point of view. They are essentially nautical in spirit, dealing exclusively with the sea and with fisher folk, and reproducing with much force and effect the rugged country and bare life of the coast. They are full of the pathos which clings to the record of half-lived lives and starved capabilities, and not wanting, too, in the occasional touches of grandeur to which such lives may rise. The reader will be reminded sometimes of the primitive customs and simple country folk among whom Miss Wilkins, in her own country, has revealed a deep under current of poetry. "The Romance of the Downs" is very much in her manner. It is a mere sketch, relating the tragic death of the three fisher brothers, to whom the heroine became successively betrothed, until, when the last was drowned, and the unhappy father was left desolate, she nursed him back to life and reason, and ended by becoming his wife. "The people on the Downs talked about it a good deal, and said it was odd of Sara to marry the old man after she had been the sweetheart of all his three sons. But the people on the Downs—and a good many others—think and speak generally as if there were only one kind of love. And in reality there are many." "The Church Ship" is the most attractive of the stories, and gives a very touching picture of the life of the fisher-folk and their manner of thought. There is the young, timid, learned pastor ashamed of his nautical knowledge and afraid of his flock; there is the Church Ship, newly restored by the fishermen, and hung up in the church in full view of all as The Seamen's Remembrance; and there are the silent, sober fishermen with their wives gathered together to hear the pastor deliver an appropriate discourse, not hoping to understand it, and unused to church, "except on very solemn occasions, when it was, so to speak, inevitable." "The preacher stood in the pulpit; he was very pale, he had spent half the night working at his sermon. The church had never been so full before, and he was quite bewildered with the thought that he must speak to all these people who had never been there before, and, perhaps, would never come again." How he began to talk of the Church's means of grace, and the Greek derivation of the expression "Ship of the Church," and how the ship turned in her chains and put all his sermon out of his head, would be too long to relate.

